# Severe Case of Intrathecal Baclofen Withdrawal: A Case Report

**DOI:** 10.7759/cureus.81141

**Published:** 2025-03-25

**Authors:** Helena Silva, Paula Barbosa, Vera Fernandes, Luís Pereira, Armanda Gomes

**Affiliations:** 1 Anesthesiology, Hospital do Divino Espírito Santo, Ponta Delgada, PRT; 2 Anesthesiology and Perioperative Medicine, Unidade Local de Saúde de São João, Porto, PRT

**Keywords:** baclofen withdrawal syndrome, gaba receptors, intrathecal baclofen, invasive mechanical ventilation, spasticity treatment

## Abstract

Baclofen is a medication that helps manage muscle spasticity by targeting gamma-aminobutyric acid B receptors in the nervous system. Discontinuing baclofen therapy generally leads to the recurrence of baseline spasticity and rigidity; however, abrupt cessation may also result in neurological, autonomic, and psychiatric symptoms. Baclofen withdrawal syndrome is one of the most serious complications of baclofen therapy, with the potential for rapid progression and significant morbidity and mortality. Prompt recognition and effective treatment are crucial. We present a severe case of intrathecal baclofen withdrawal following the sudden cessation of therapy, emphasizing the critical need for careful monitoring and early intervention to prevent serious complications.

## Introduction

Baclofen, a gamma-aminobutyric acid (GABA) B receptor agonist, is widely used to manage muscle spasticity [1]. It effectively reduces muscle tone and spasticity by inhibiting the release of excitatory neurotransmitters, thereby suppressing the overactive stretch reflex [1,2]. While generally well tolerated, abrupt discontinuation can trigger a range of withdrawal symptoms [3]. Typically, baclofen withdrawal presents as a return of the patient’s baseline spasticity and rigidity [3,4]. However, in more severe cases, sudden cessation may lead to a constellation of neurological, autonomic, and psychiatric symptoms that mimic autonomic dysreflexia, malignant hyperthermia, or neuroleptic malignant syndrome [2-4]. If left untreated, baclofen withdrawal syndrome (BWS) can progress to multi-organ failure and death.

BWS is the most concerning complication of baclofen therapy. Due to its symptom overlap with other acute conditions, along with its potential for rapid progression and high morbidity and mortality rates, prompt recognition and timely treatment are essential [4,5]. The most effective management involves the reinitiation or supplementation of baclofen, either orally or via intrathecal administration, to restore therapeutic levels in the CNS [4,6].

Here, we present a severe case of intrathecal baclofen (ITB) withdrawal following the abrupt cessation of therapy. This case highlights the critical need for careful monitoring and the potential for rapid clinical deterioration, emphasizing the importance of early recognition and intervention to prevent serious complications.

## Case presentation

A 37-year-old male with C4 tetraplegia (ASIA grade C) secondary to a cervical spinal cord injury following a traffic accident in 2008 initially underwent spasticity management with oral baclofen (25 mg every eight hours), which proved unsuccessful. In 2009, an ITB pump was implanted, with drug titration to 203 mcg/day, providing significant improvement and allowing the patient to regain functional independence while maintaining regular follow-up at the chronic pain unit.

In 2022, the patient was admitted to the hospital for ITB pump replacement due to battery failure. The procedure was uneventful, and he was discharged three days later with the pump delivering 210 mcg/day. Frequent follow-ups at the chronic pain unit to monitor neurological function, spasticity, and surgical wounds were uneventful. However, 15 days post-discharge, he developed erythema at the pump implantation site without other associated symptoms. Despite conservative management, the erythema persisted. After three months of follow-up, he was admitted to the hospital due to wound dehiscence. Oral baclofen replacement therapy (25 mg every eight hours) was initiated, and the ITB pump was removed intraoperatively. Cultures from the wound and cerebrospinal fluid were obtained, and empirical antibiotics were started.

Five days following ITB pump removal, the patient became somnolent and experienced periods of confusion, worsening spasticity, fever, and hypertension. BWS was suspected, and he was admitted to the ICU. Upon admission, oral baclofen was increased to 20 mg every six hours, and a 50 mcg ITB single-shot bolus was administered. Despite improved spasticity, his level of consciousness continued to deteriorate, accompanied by myoclonic-like movements, requiring deep sedation and intubation. Due to kidney impairment, oral baclofen dosing was optimized to 25 mg every eight hours.

Over the following days, multiple attempts to reduce sedation were ineffective due to persistent psychomotor agitation. A multidisciplinary team, including specialists in chronic pain and intensive care, decided to place an intrathecal catheter on the 13th day of ICU stay and reinitiate ITB administration. A total of two 50 mcg boluses were administered four hours apart. Subsequently, the patient’s level of consciousness significantly improved, leading to reduced sedation requirements. He recovered spontaneous ventilation by the 19th day of ICU stay and was discharged after 46 days, scheduled to continue outpatient physical rehabilitation and follow-up at the chronic pain unit.

Unexpectedly, he exhibited marked clinical improvement, including a reduction in spasticity compared to his baseline condition. Following this episode, his spasticity remained well managed with oral baclofen (25 mg every eight hours), and there was no indication for ITB pump replacement.

## Discussion

Baclofen, a GABA B receptor agonist, is widely used to manage muscle spasticity, particularly in conditions such as spinal cord injury, multiple sclerosis, cerebral palsy, traumatic brain injury, and cardiovascular strokes [1,2]. It reduces the release of excitatory neurotransmitters by enhancing presynaptic GABA B receptor activation, leading to the suppression of spinal reflexes and a reduction in muscle spasms, thereby alleviating associated pain and discomfort [3-5].

Baclofen can be administered orally or intrathecally, with the latter offering several therapeutic advantages, including higher drug concentrations directly at the spinal cord, which are less achievable with oral administration [5-7]. However, ITB delivery systems are susceptible to mechanical malfunctions, programming errors, catheter obstructions or kinks, dislodgement or leakage, battery depletion, and unrecognized declines in pump reservoir drug levels [4,8].

A significant complication associated with baclofen therapy, particularly when intrathecal administration is suddenly discontinued or interrupted, is BWS [9,10]. This syndrome is characterized by a loss of GABAergic inhibition, leading to an exaggerated excitatory response in the CNS, which can present with severe symptoms such as generalized spasticity, muscle rigidity, seizures, autonomic dysregulation, and altered mental status [3-5]. These symptoms are often nonspecific and may overlap with other conditions, including meningitis, sepsis, malignant hyperthermia, neuroleptic malignant syndrome, and autonomic dysreflexia [4,11]. Given this overlap, early recognition is crucial to prevent potentially life-threatening complications.

The primary treatment for BWS is the reintroduction of baclofen to restore CNS levels as quickly as possible [6]. Both oral and ITB can be used for replacement therapy, but intrathecal administration plays a critical role in this process [4,6]. Direct intrathecal delivery achieves therapeutic cerebrospinal fluid concentrations with plasma levels 100 times lower than those associated with oral administration, which is essential for effectively managing severe withdrawal symptoms, particularly in patients with compromised CNS absorption or those who have experienced rapid or prolonged drug cessation [4,5]. Furthermore, baclofen’s water solubility limits its ability to cross the blood-brain barrier (BBB) when administered orally, making it inadequate for managing acute withdrawal [5,6].

In the case presented, the patient experienced severe withdrawal symptoms following the sudden cessation of ITB therapy. Despite initiating oral baclofen at an appropriate dosage, symptoms worsened, underscoring the inability of oral administration to restore therapeutic CNS concentrations quickly enough. As the patient’s condition deteriorated, intensive care measures, including deep sedation and mechanical ventilation, became necessary. A single dose of ITB provided only partial relief of spasticity and failed to reverse neurological deterioration completely. Notably, significant symptom improvement occurred only after six days, following the cumulative administration of 100 mcg. The pharmacological limitations of oral baclofen, such as its short half-life and poor BBB penetration, render it an imperfect substitute for ITB. Additionally, oral baclofen absorption may be impaired in critically ill patients, and no reliable oral-to-intrathecal conversion dose exists, further highlighting the critical role of intrathecal administration in managing severe baclofen withdrawal in patients previously receiving high-dose ITB [5,6].

Similar cases have been reported in the literature. Greenberg et al. and Mohammed and Hussain described patients who developed severe withdrawal after abrupt discontinuation of ITB and required both oral and intrathecal reintroduction to stabilize their conditions [12,13]. Other reports by Ross et al. and Douglas et al. demonstrated that the rapid restoration of therapeutic baclofen levels through intrathecal administration is essential for reversing the severe neurological and autonomic symptoms associated with withdrawal [9,14]. These cases further support our findings, where delayed response to oral baclofen necessitated intrathecal reintroduction to achieve clinical recovery.

A literature review describes cases of ITB withdrawal requiring intubation [13,15]. This corresponds to our case, in which the patient required mechanical ventilation and deep sedation due to worsening neurological function. Improvement was observed only after therapeutic baclofen levels were reestablished via intrathecal administration. A notable distinction in our case was the slower recovery - six days after intrathecal dosage optimization, totaling 19 days - compared to previously reported cases, where patients typically showed clinical improvement within two to three days following ITB reintroduction. The delayed recovery in our patient could be attributed to several factors, including the severity of withdrawal, underlying medical conditions, and potential delays in reestablishing effective baclofen concentrations.

Our case aligns with others in the literature, highlighting the complexity of managing severe baclofen withdrawal and the critical importance of timely and appropriate interventions. This includes early intubation, prompt ITB reintroduction, and intensive care management, particularly for critically ill patients.

## Conclusions

BWS remains a significant concern, particularly following the discontinuation of ITB. Restoring therapeutic baclofen levels through oral administration alone is challenging and well documented, whereas intrathecal administration has proven to be more effective, especially in critically ill patients. Although baclofen withdrawal is relatively rare, its severity underscores the urgent need for further clinical research to better understand and optimize management strategies, particularly for patients requiring intensive care or mechanical ventilation. This case highlights the critical importance of timely and appropriate interventions in managing severe withdrawal symptoms and improving patient outcomes.
